# Supplementary material for: Intrahost SARS-CoV-2 k-mer Identification Method (iSKIM) for Rapid Detection of Mutations of Concern Reveals Emergence of Global Mutation Patterns
Source: Viruses. 2022 Sep 27;14(10):2128. doi: 10.3390/v14102128 (PMC9609618; doi:10.3390/v14102128)

Mutations Found in the Alpha Variant (B.1.1.7 lineage)

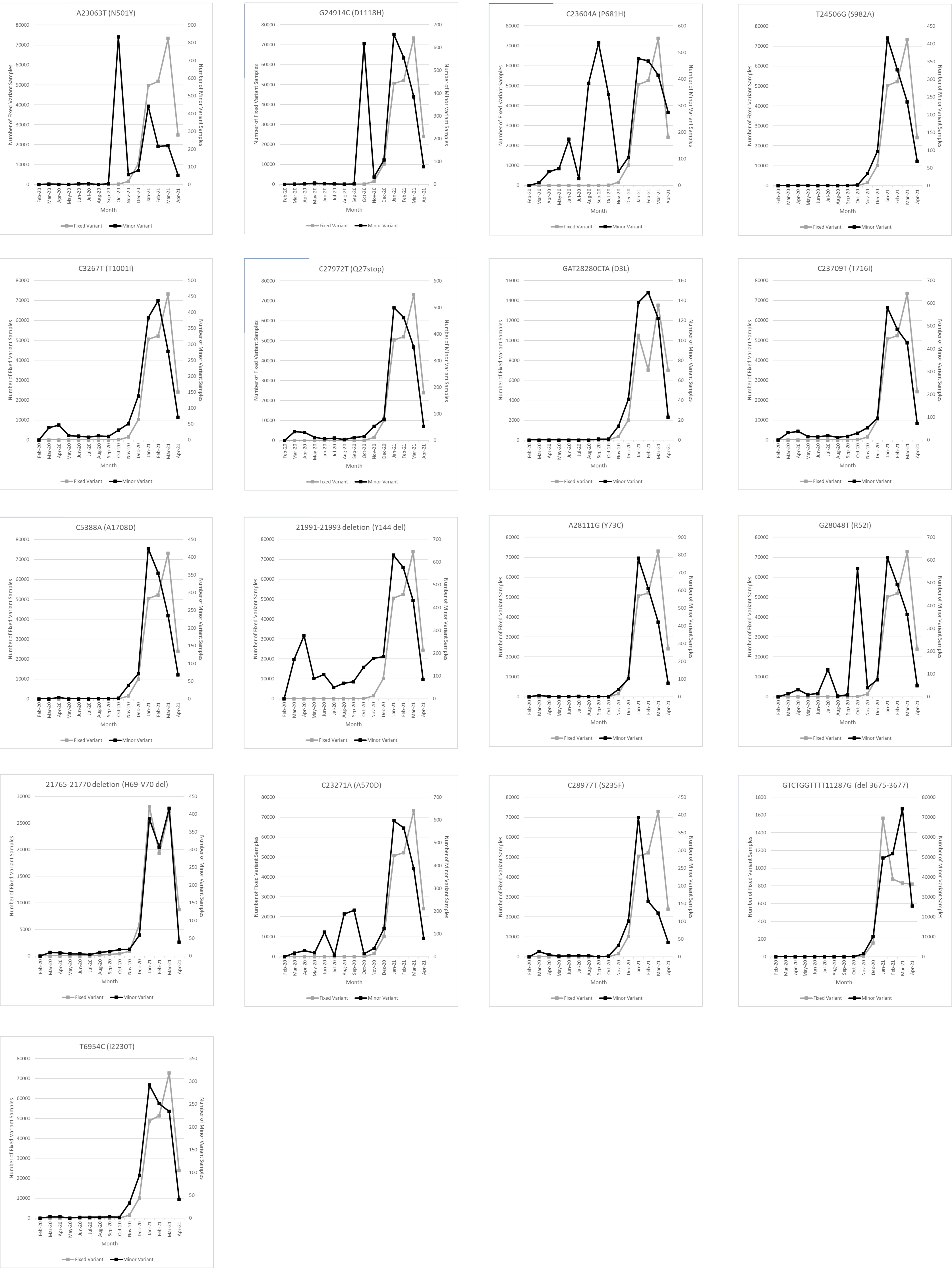

Mutations Found in the Beta Variant (B.1.351 Lineage)

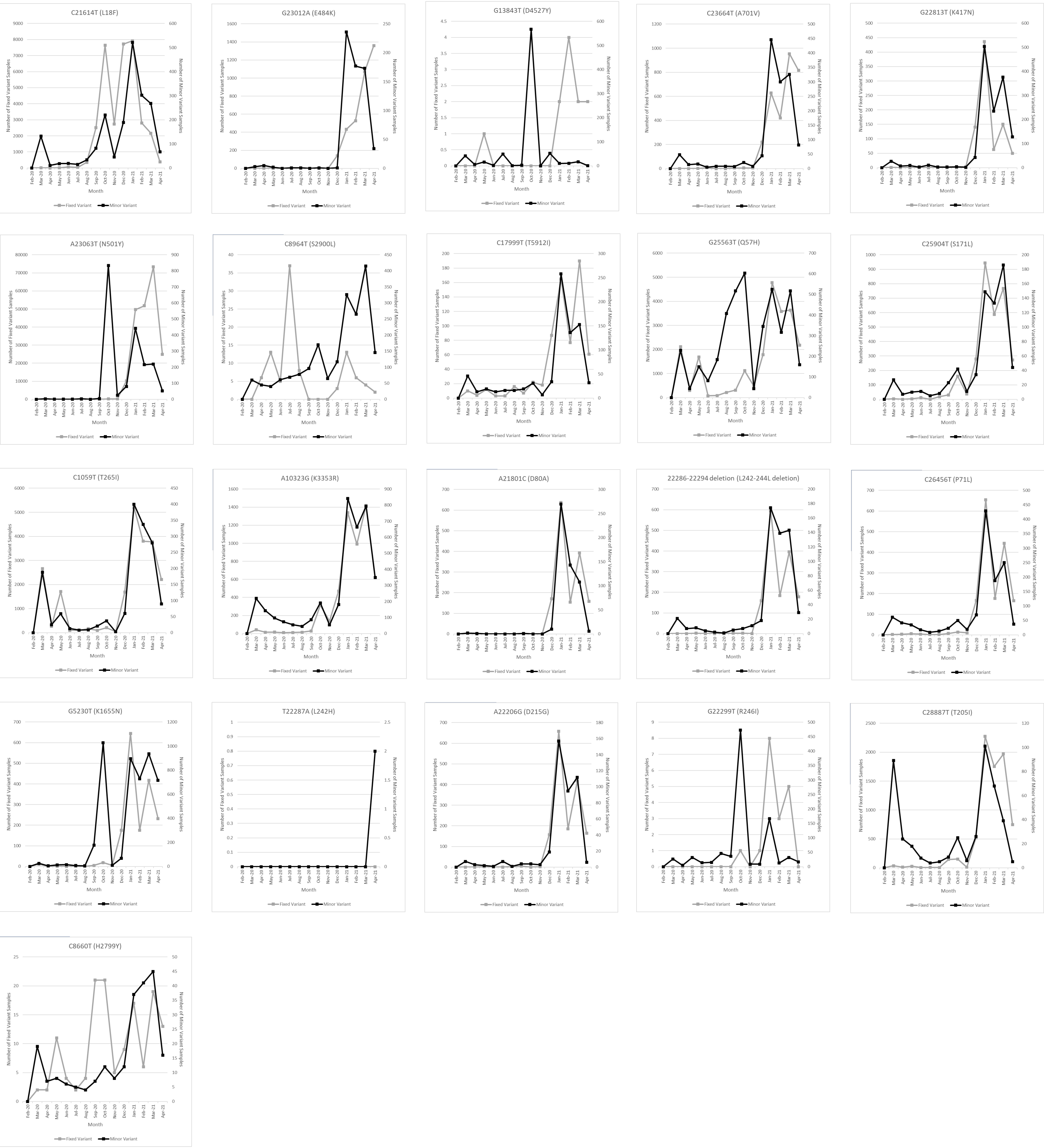

Mutations Found in the Gamma Variant (P.1 Lineage)

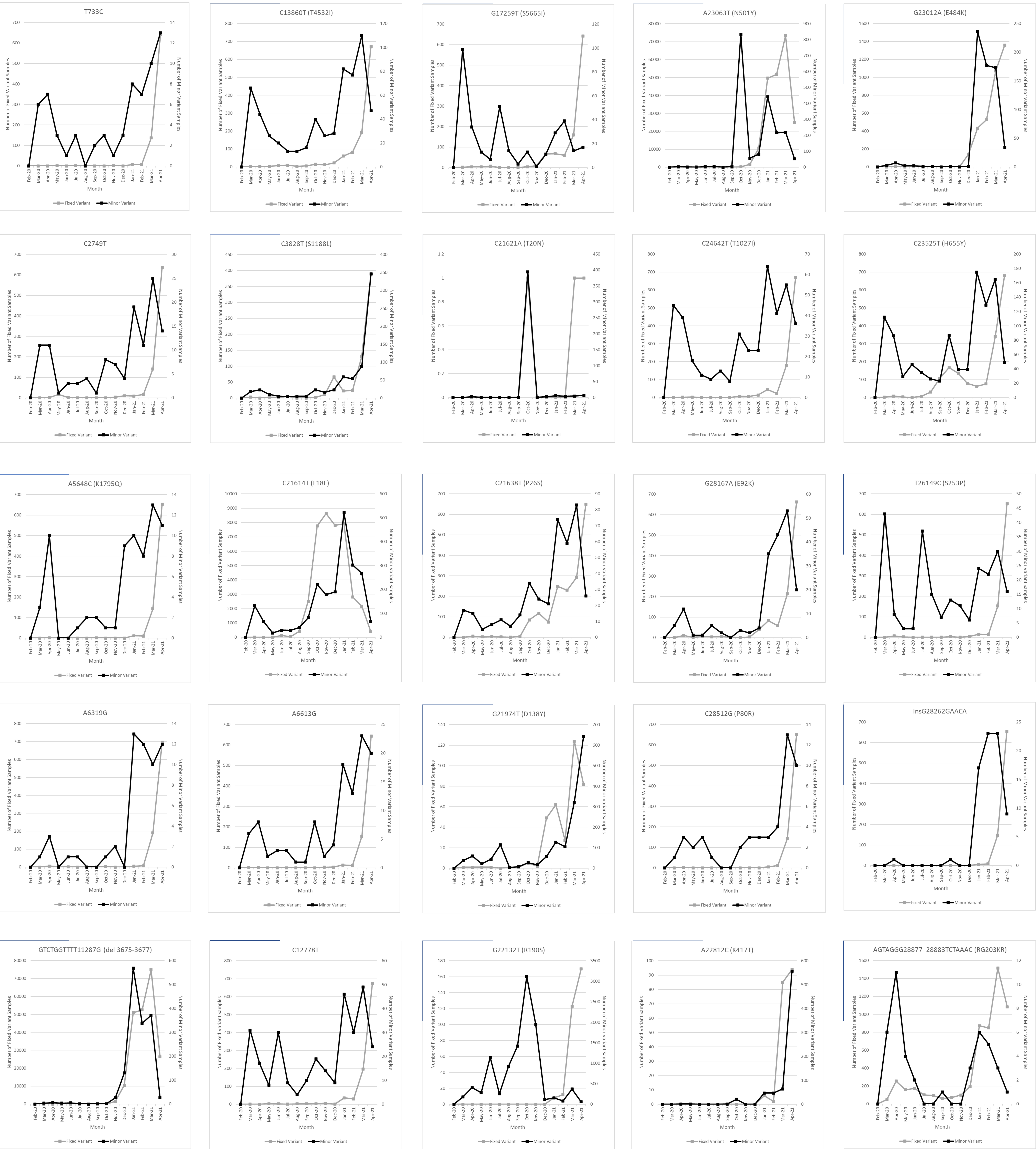

Mutations Found in the Delta Variant (B.1.617.2 Lineage)

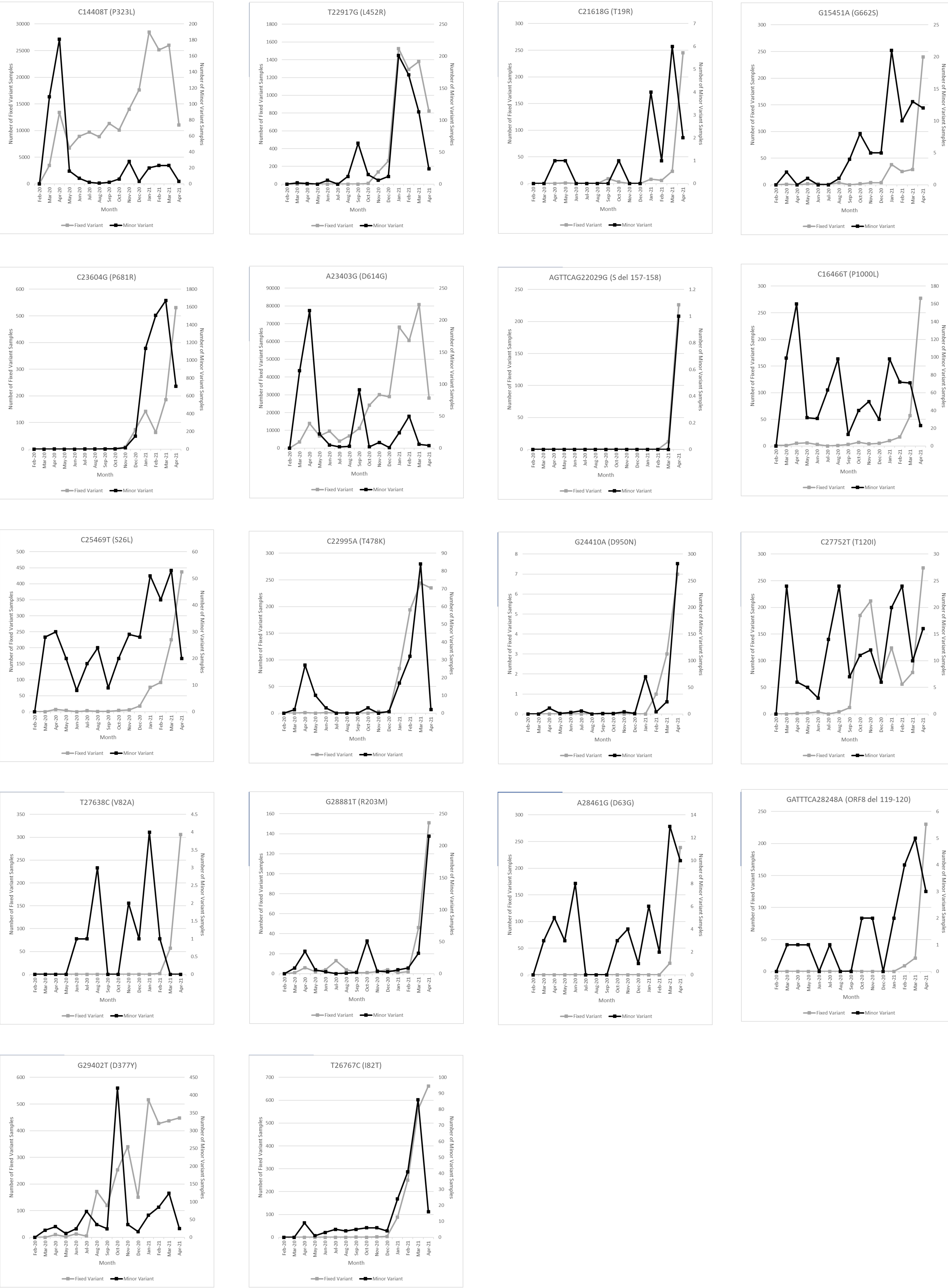

Mutations Found in the Epsilon Variant (B.1.429 lineage)

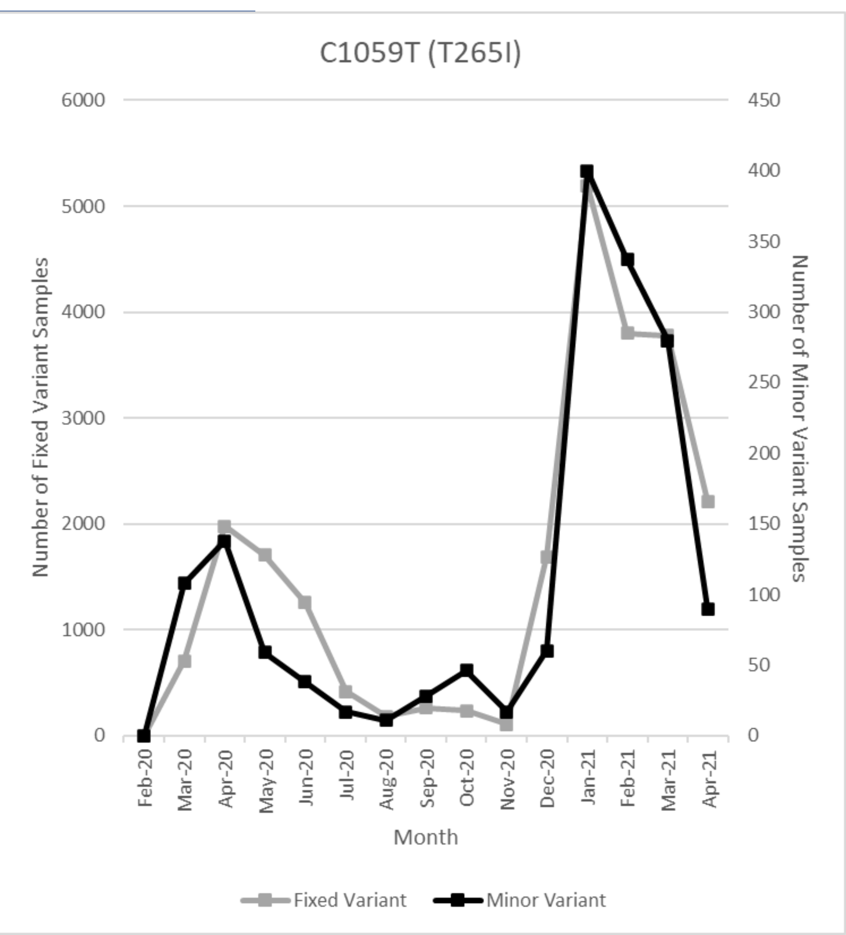

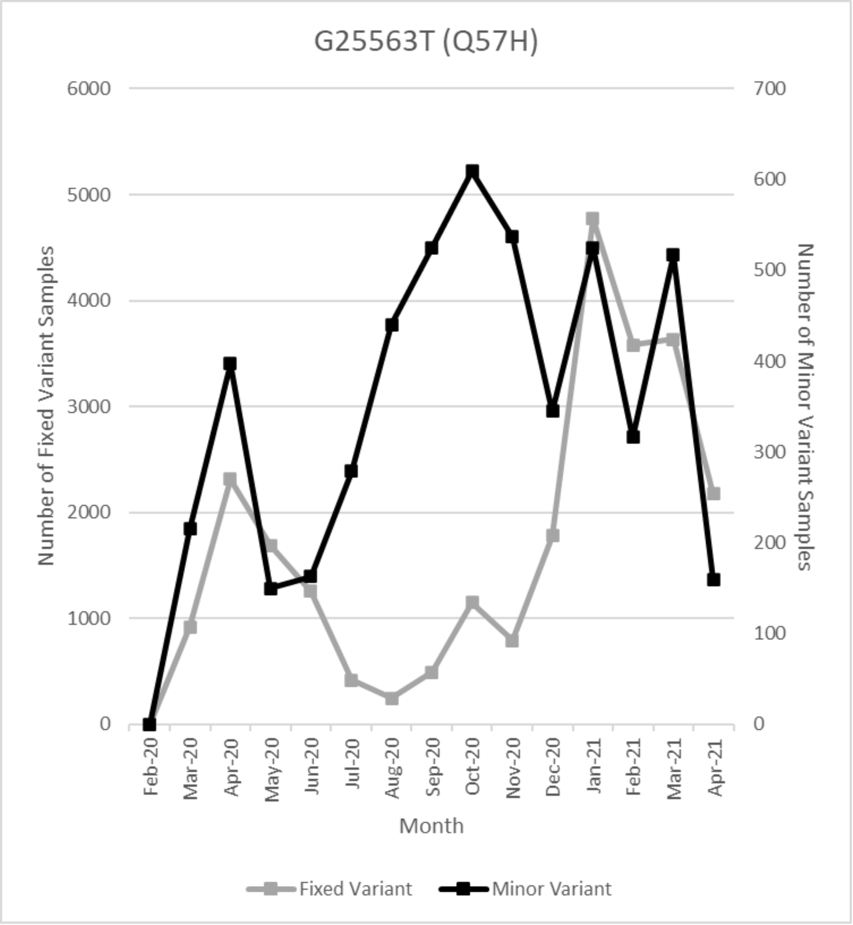

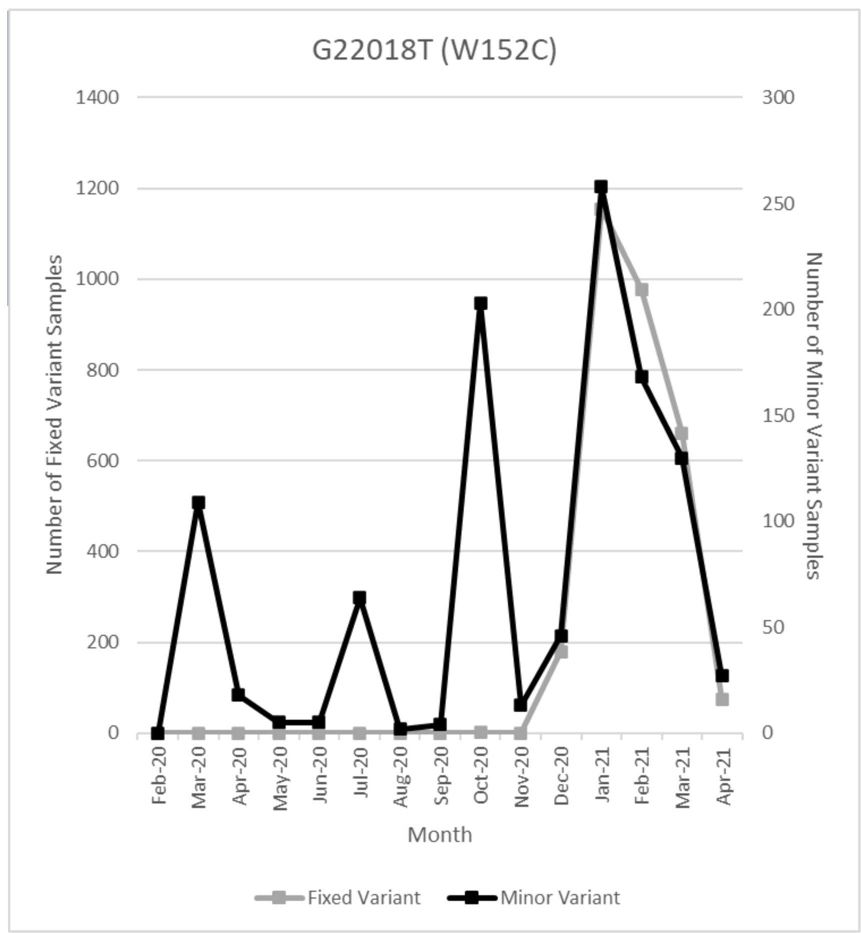

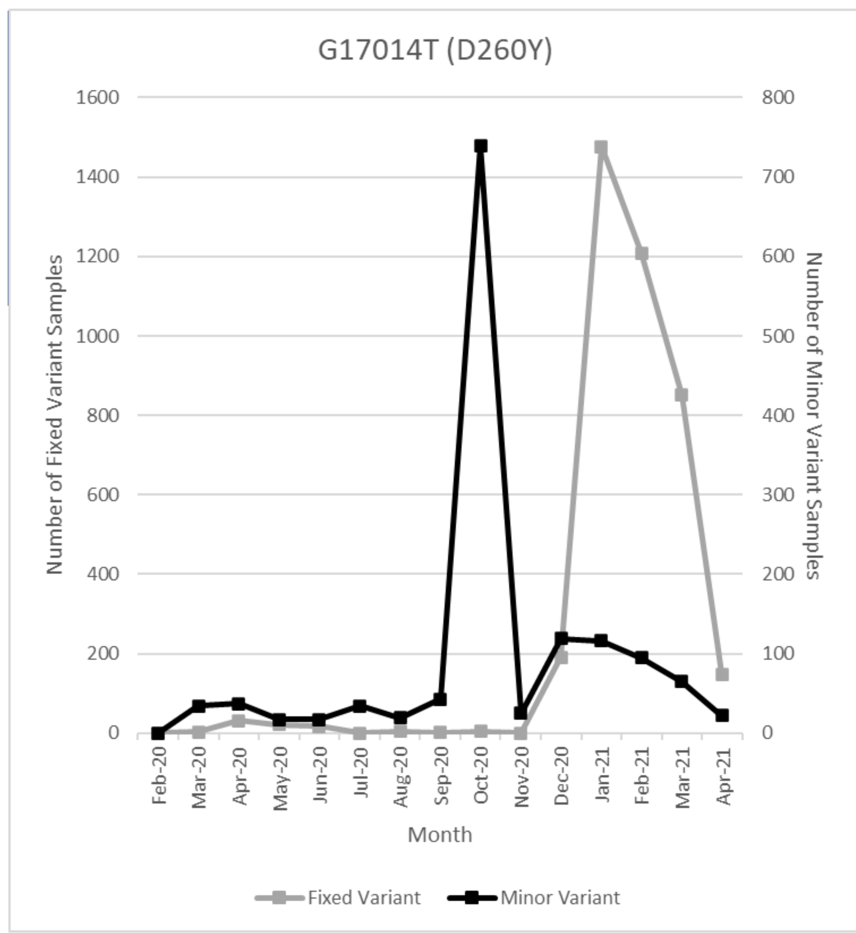

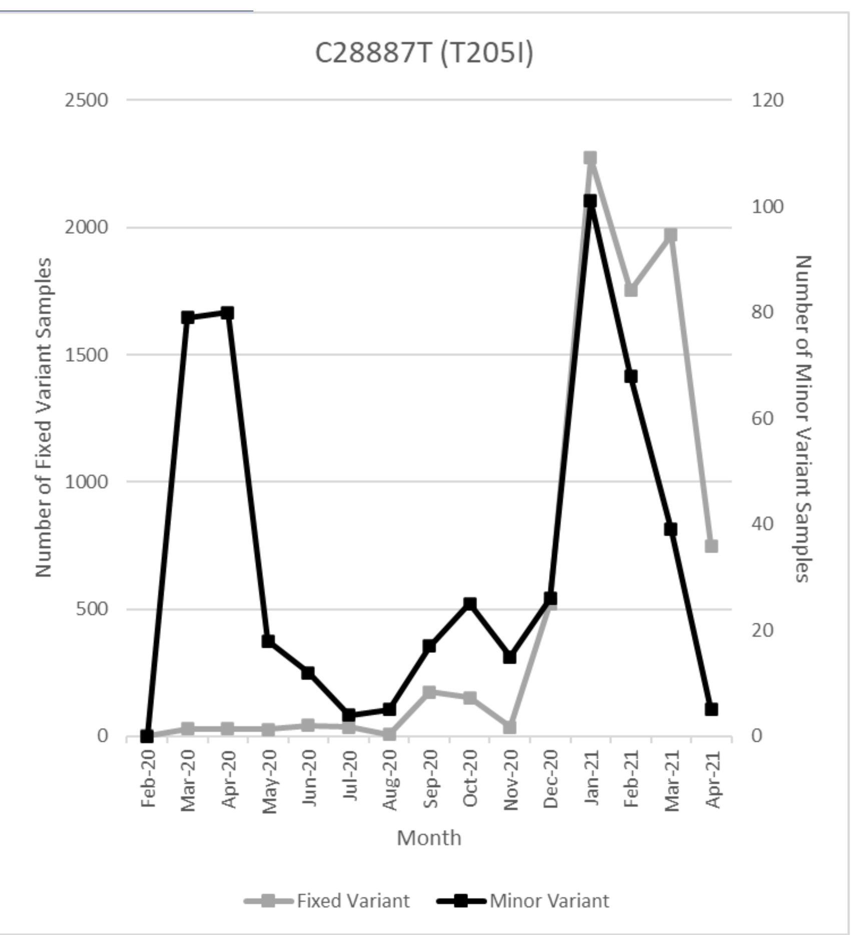

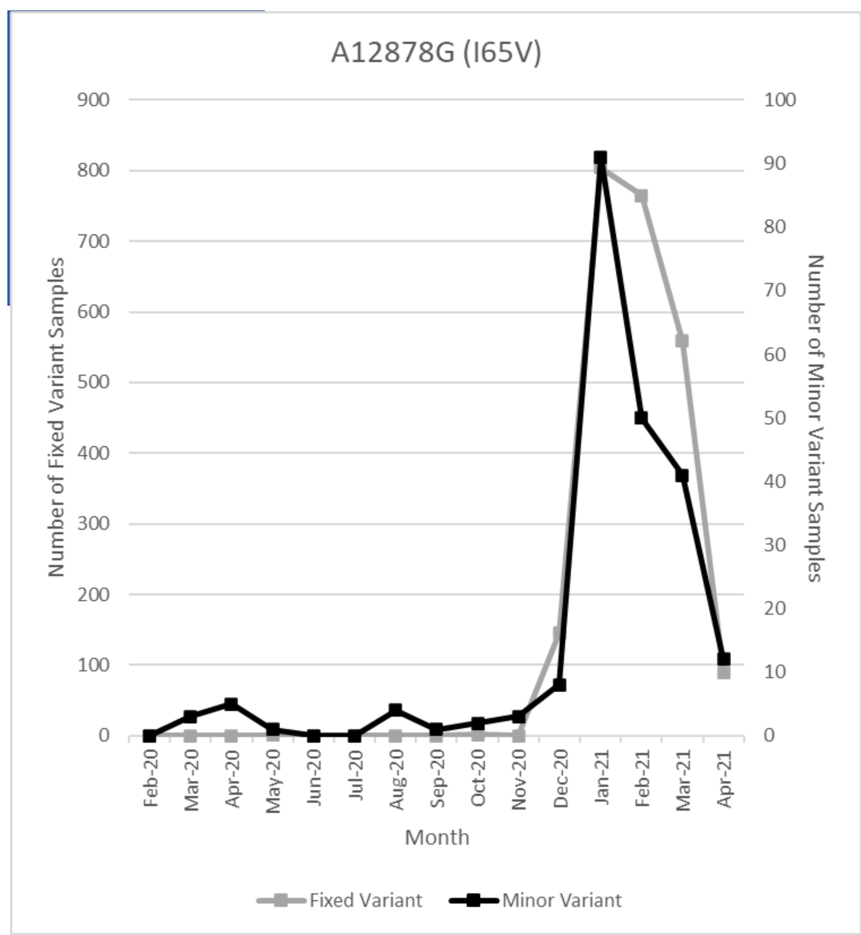

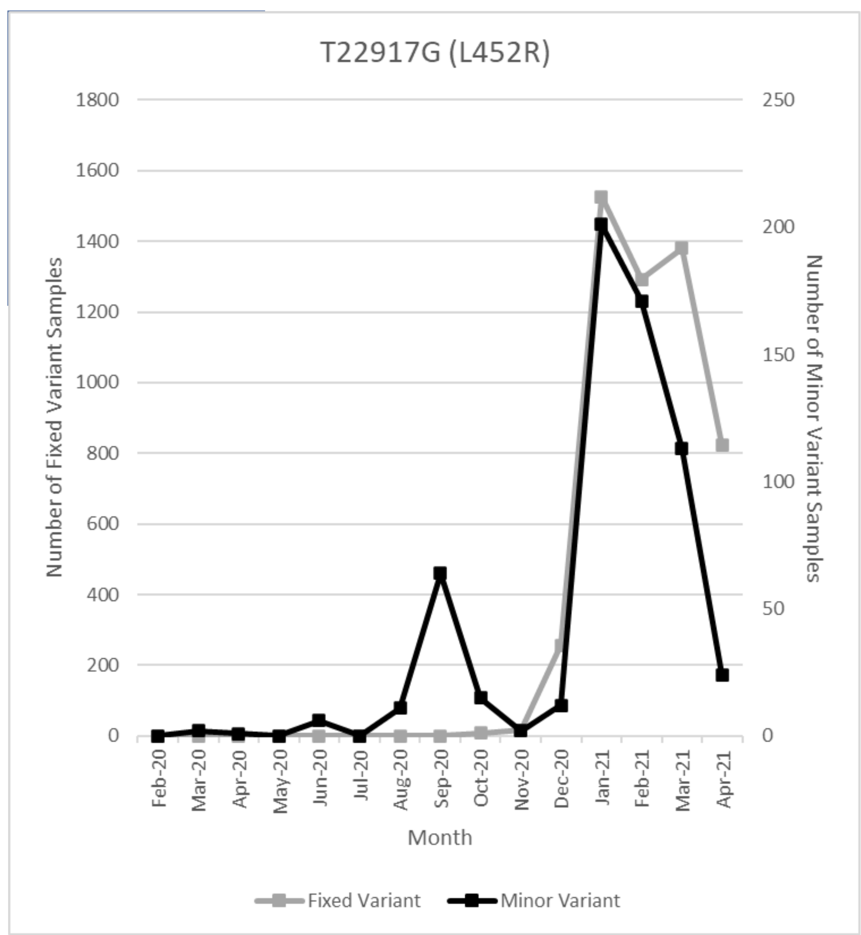

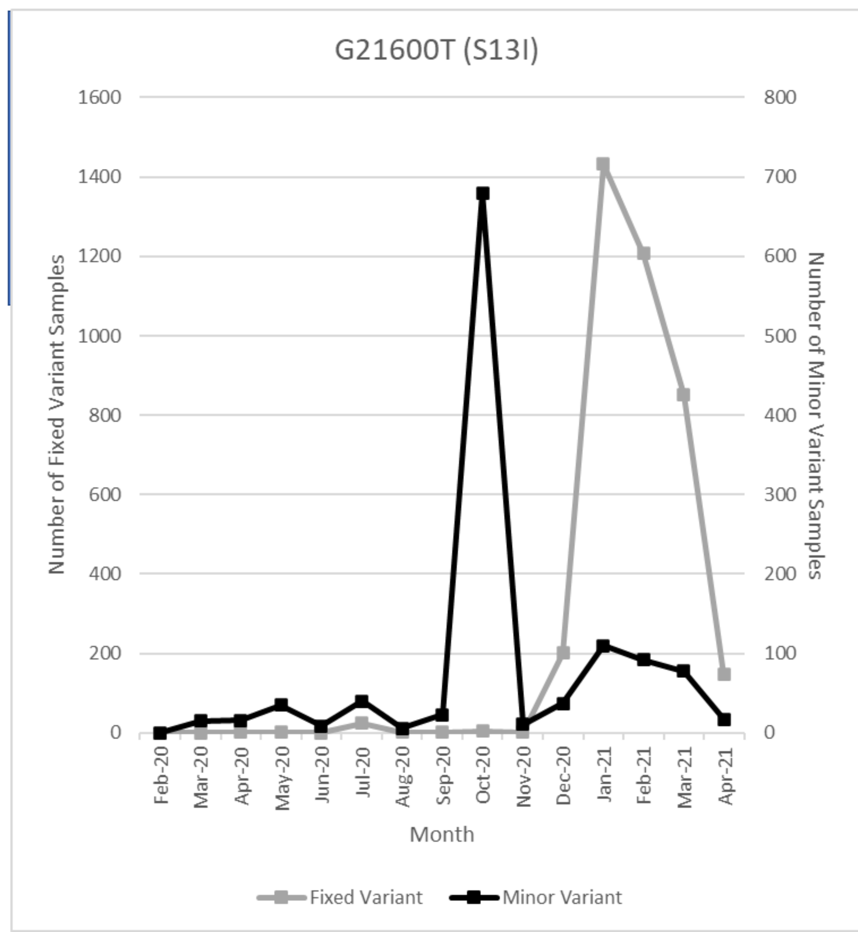

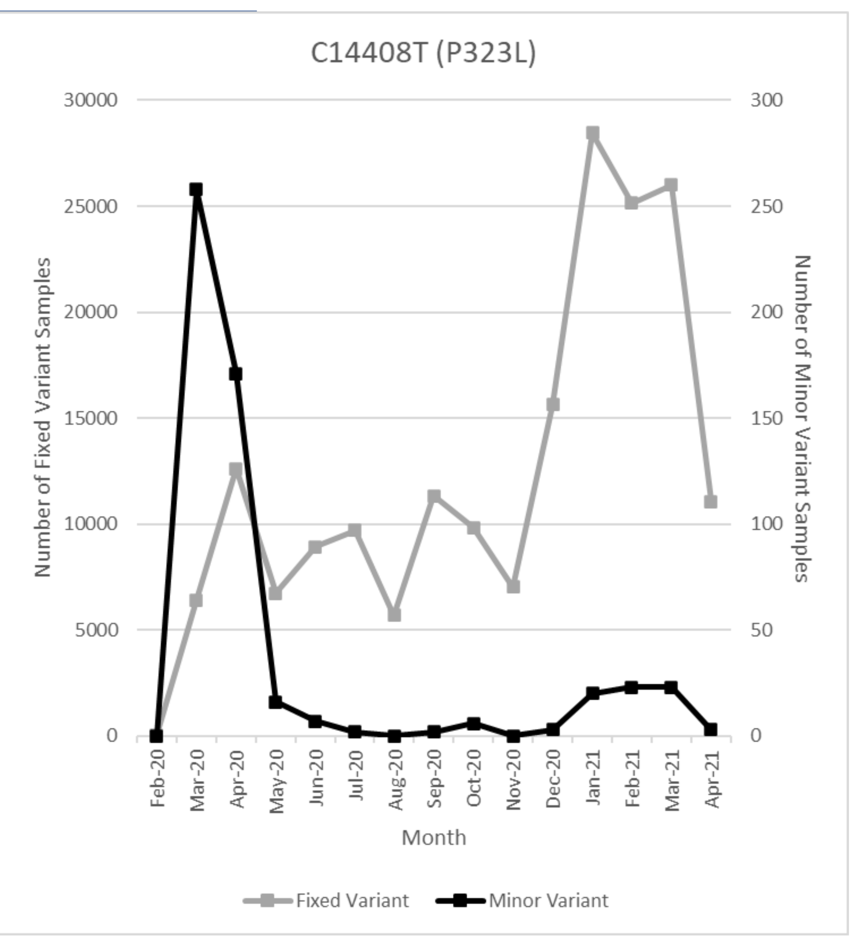

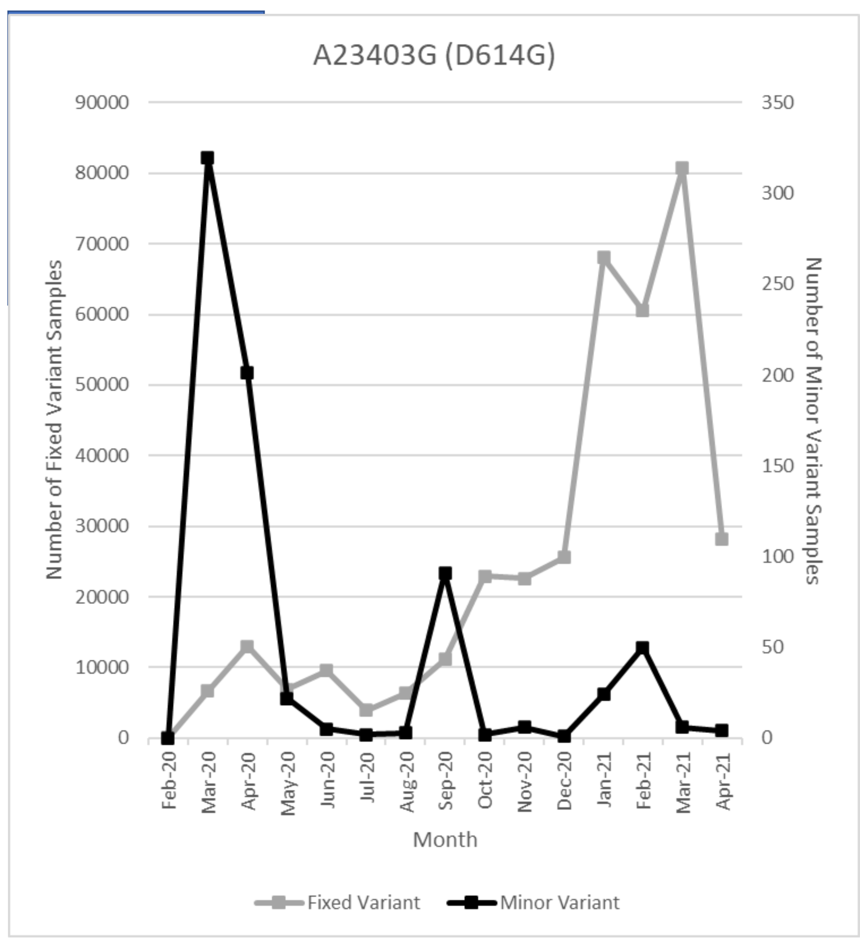

Mutations Found in the Iota Variant (B.1.526 lineage)

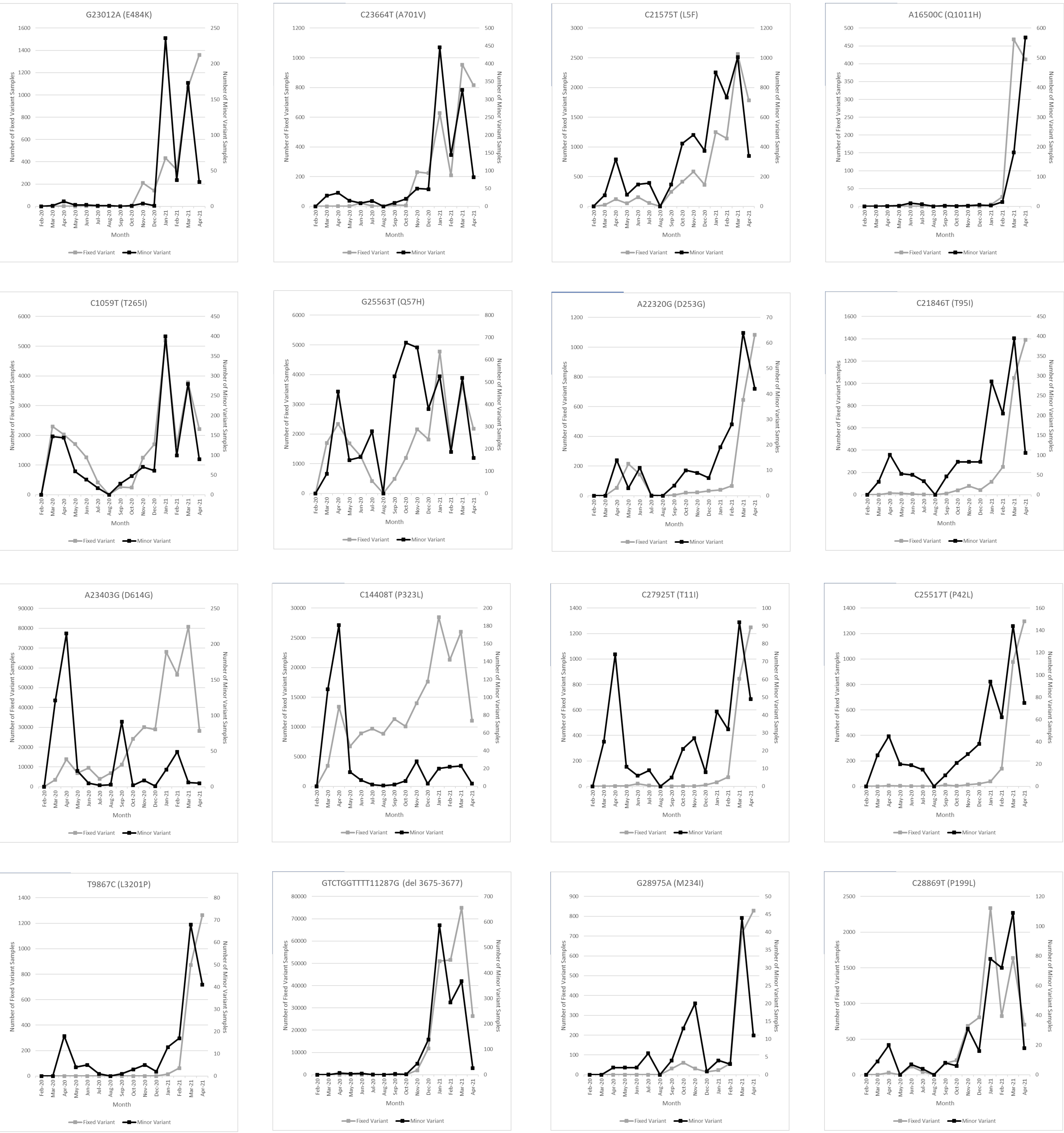

Mutations Found in the Kappa Variant (B.1.617.1 Lineage)

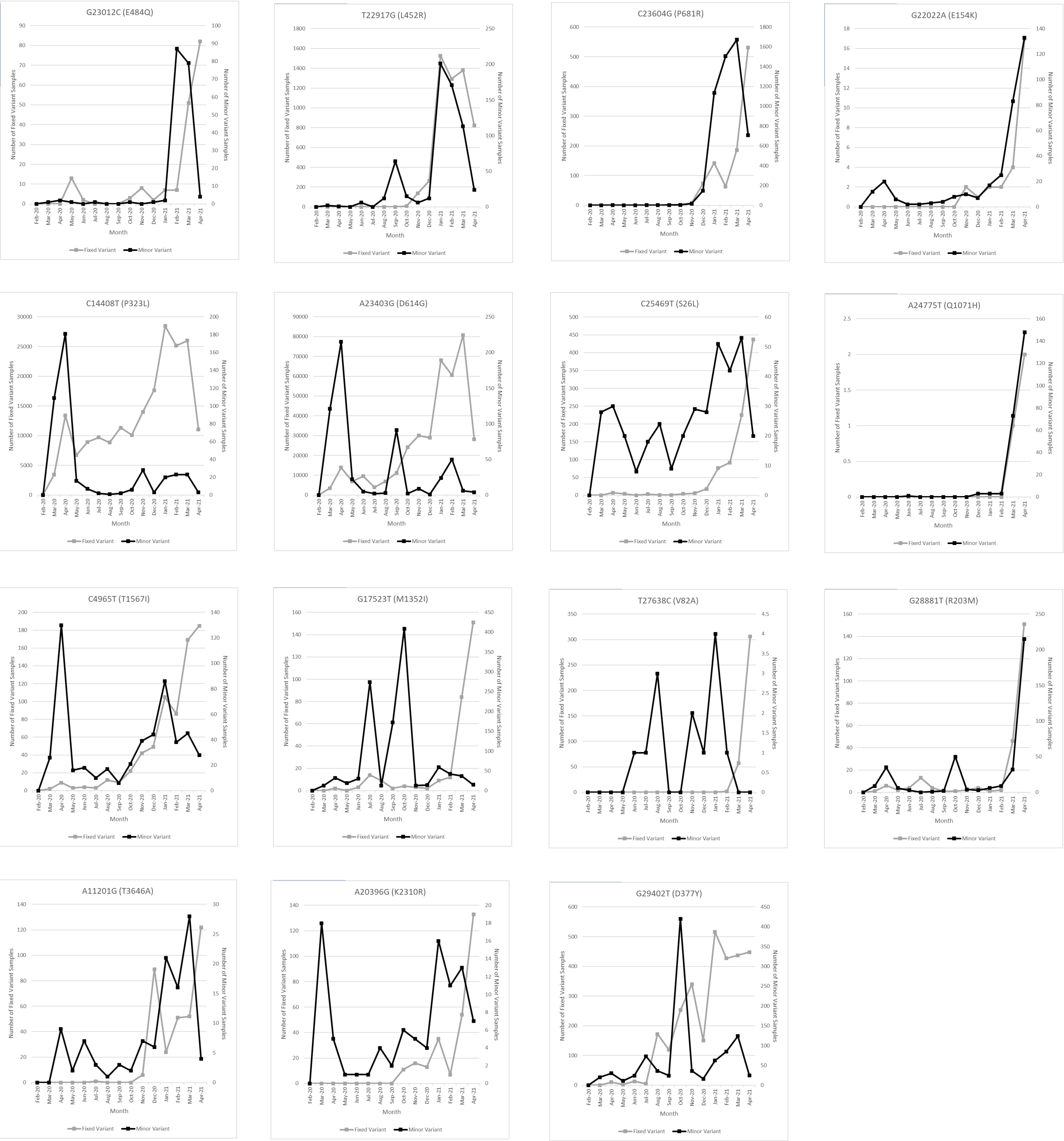

Supplement: Supplementary file 1 [file viruses-14-02128-s001.zip › Supplementary_Files/FigureS1.pdf]
