# Supplementary material for: Intrahost SARS-CoV-2 k-mer Identification Method (iSKIM) for Rapid Detection of Mutations of Concern Reveals Emergence of Global Mutation Patterns
Source: Viruses. 2022 Sep 27;14(10):2128. doi: 10.3390/v14102128 (PMC9609618; doi:10.3390/v14102128)
Supplement: Supplementary file 1 [file viruses-14-02128-s001.zip › Supplementary_Files/TableS4.pdf]

We gratefully acknowledge the following Authors from the Originating laboratories responsible for obtaining the specimens, as well as the Submitting laboratories where the genome data were generated and shared via GISAID, on which this research is based.

All Submitters of data may be contacted directly via www.gisaid.org

Authors are sorted alphabetically.

Acknowledgement EPI_SET Identifier: EPI_SET_20220218wq

Accession ID	Originating Laboratory	Submitting Laboratory	Authors
EPI_ISL_611114, EPI_ISL_611142, EPI_ISL_611281, EPI_ISL_625032	Lighthouse Lab in Alderley Park	Wellcome Sanger Institute for the COVID-19 Genomics UK (COG-UK) consortium	Cordelia Langford; David K. Jackson; Dominic Kwiatkowski; Ewan Harrison; Ian Johnston; Jacquelyn Wynn; John Sillitoe on behalf of the Wellcome Sanger Institute COVID-19 Surveillance Team (http://www.sanger.ac.uk/covid-team); Mairead Hyland; Roberto Amato; Sonia Goncalves; The Lighthouse Lab in Alderley Park and Alex Alderton
EPI_ISL_643565, EPI_ISL_672920, EPI_ISL_672966, EPI_ISL_673063, EPI_ISL_673100, EPI_ISL_673124, EPI_ISL_673133, EPI_ISL_673200			
see above	Lighthouse Lab in Cambridge	Wellcome Sanger Institute for the COVID-19 Genomics UK (COG-UK) Consortium	Cordelia Langford; David K. Jackson; Dominic Kwiatkowski; Ewan Harrison; Ian Johnston; John Sillitoe on behalf of the Wellcome Sanger Institute COVID-19 Surveillance Team; Rob Howes; Roberto Amato; Sonia Goncalves; The Lighthouse Lab in Cambridge and Alex Alderton
EPI_ISL_608679	Lighthouse Lab in Cambridge	Wellcome Sanger Institute for the COVID-19 Genomics UK (COG-UK) consortium	Cordelia Langford; David K. Jackson; Dominic Kwiatkowski; Ewan Harrison; Ian Johnston; John Sillitoe on behalf of the Wellcome Sanger Institute COVID-19 Surveillance Team; Rob Howes; Roberto Amato; Sonia Goncalves; The Lighthouse Lab in Cambridge and Alex Alderton
EPI_ISL_649688, EPI_ISL_649731, EPI_ISL_649739, EPI_ISL_662675, EPI_ISL_662677, EPI_ISL_662688, EPI_ISL_662704, EPI_ISL_662747, EPI_ISL_662754, EPI_ISL_662764, EPI_ISL_662769, EPI_ISL_662771, EPI_ISL_662789, EPI_ISL_662803, EPI_ISL_662823, EPI_ISL_662824, EPI_ISL_662834, EPI_ISL_662840, EPI_ISL_662849, EPI_ISL_662850, EPI_ISL_662875, EPI_ISL_662890, EPI_ISL_662897, EPI_ISL_662900, EPI_ISL_662901, EPI_ISL_662902, EPI_ISL_662905, EPI_ISL_662906, EPI_ISL_662915, EPI_ISL_662916, EPI_ISL_662917, EPI_ISL_662918, EPI_ISL_662920, EPI_ISL_662922, EPI_ISL_662923, EPI_ISL_662926, EPI_ISL_662927, EPI_ISL_662929, EPI_ISL_662931, EPI_ISL_662932, EPI_ISL_662934, EPI_ISL_662936, EPI_ISL_662938, EPI_ISL_662940, EPI_ISL_662943, EPI_ISL_662944, EPI_ISL_662946, EPI_ISL_662947, EPI_ISL_662950, EPI_ISL_662951, EPI_ISL_662954, EPI_ISL_662963, EPI_ISL_662966, EPI_ISL_662968, EPI_ISL_662975, EPI_ISL_662976, EPI_ISL_662984, EPI_ISL_662985, EPI_ISL_662986, EPI_ISL_662990, EPI_ISL_662992, EPI_ISL_662993, EPI_ISL_662994, EPI_ISL_662996, EPI_ISL_663000, EPI_ISL_663008, EPI_ISL_663010, EPI_ISL_663011, EPI_ISL_663012, EPI_ISL_663013, EPI_ISL_663016, EPI_ISL_663018, EPI_ISL_663020, EPI_ISL_663021, EPI_ISL_663023, EPI_ISL_663024, EPI_ISL_663025, EPI_ISL_663027, EPI_ISL_663031, EPI_ISL_663032, EPI_ISL_663036, EPI_ISL_663037, EPI_ISL_663038, EPI_ISL_663040, EPI_ISL_663042, EPI_ISL_663047, EPI_ISL_663049, EPI_ISL_663050, EPI_ISL_663051, EPI_ISL_663053, EPI_ISL_663054, EPI_ISL_663058, EPI_ISL_663060, EPI_ISL_663061, EPI_ISL_663065, EPI_ISL_663066, EPI_ISL_663070, EPI_ISL_663071, EPI_ISL_663075, EPI_ISL_663077, EPI_ISL_663078, EPI_ISL_663080, EPI_ISL_663081, EPI_ISL_663082, EPI_ISL_663089, EPI_ISL_663093, EPI_ISL_663099, EPI_ISL_663101, EPI_ISL_663103, EPI_ISL_663105, EPI_ISL_663107, EPI_ISL_663109, EPI_ISL_663110, EPI_ISL_663113, EPI_ISL_663115, EPI_ISL_663118, EPI_ISL_663121, EPI_ISL_663124, EPI_ISL_663129, EPI_ISL_663130, EPI_ISL_663131, EPI_ISL_663133, EPI_ISL_663135, EPI_ISL_663139, EPI_ISL_663140, EPI_ISL_663142, EPI_ISL_663145, EPI_ISL_663146, EPI_ISL_663147, EPI_ISL_663149, EPI_ISL_663151, EPI_ISL_663158, EPI_ISL_663160, EPI_ISL_663167, EPI_ISL_663168, EPI_ISL_663171, EPI_ISL_663172, EPI_ISL_663173, EPI_ISL_663174, EPI_ISL_663175, EPI_ISL_663177, EPI_ISL_663179, EPI_ISL_663181, EPI_ISL_663185, EPI_ISL_663189, EPI_ISL_663191, EPI_ISL_663192, EPI_ISL_663195, EPI_ISL_663201, EPI_ISL_663202, EPI_ISL_663203, EPI_ISL_663204, EPI_ISL_676446, EPI_ISL_676451, EPI_ISL_676461, EPI_ISL_676462, EPI_ISL_676464, EPI_ISL_676465, EPI_ISL_676467, EPI_ISL_676469, EPI_ISL_676470, EPI_ISL_676472, EPI_ISL_676473, EPI_ISL_676475, EPI_ISL_676477, EPI_ISL_676482, EPI_ISL_721562			
see above	Lighthouse Lab in Glasgow	Wellcome Sanger Institute for the COVID-19 Genomics UK (COG-UK) Consortium	Anna Dominiczak and Alex Alderton; Carol Clugston; Cordelia Langford; David Gray; David K. Jackson; Dominic Kwiatkowski; Ewan Harrison; Harper VanSteenhouse; Ian Johnston; John Sillitoe on behalf of the Wellcome Sanger Institute COVID-19 Surveillance Team; John Sillitoe on behalf of the Wellcome Sanger Institute COVID-19 Surveillance Team (http://www.sanger.ac.uk/covid-team); Roberto Amato; Sonia Goncalves; Yumi Kasai
EPI_ISL_611477, EPI_ISL_625328, EPI_ISL_633477, EPI_ISL_634070, EPI_ISL_634072, EPI_ISL_634075, EPI_ISL_634097, EPI_ISL_634114, EPI_ISL_634117, EPI_ISL_634135, EPI_ISL_634207, EPI_ISL_634224, EPI_ISL_634234, EPI_ISL_634235, EPI_ISL_634240			
see above	Lighthouse Lab in Glasgow	Wellcome Sanger Institute for the COVID-19 Genomics UK (COG-UK) consortium	Anna Dominiczak and Alex Alderton; Carol Clugston; Cordelia Langford; David Gray; David K. Jackson; Dominic Kwiatkowski; Ewan Harrison; Harper VanSteenhouse; Ian Johnston; John Sillitoe on behalf of the Wellcome Sanger Institute COVID-19 Surveillance Team; John Sillitoe on behalf of the Wellcome Sanger Institute COVID-19 Surveillance Team (http://www.sanger.ac.uk/covid-team); Roberto Amato; Sonia Goncalves; Yumi Kasai
EPI_ISL_598249, EPI_ISL_598305, EPI_ISL_598330, EPI_ISL_598331, EPI_ISL_598408, EPI_ISL_598433, EPI_ISL_598606, EPI_ISL_598638, EPI_ISL_598667, EPI_ISL_598821, EPI_ISL_609230, EPI_ISL_609267, EPI_ISL_609302			
see above	Lighthouse Lab in Milton Keynes	Wellcome Sanger Institute for the COVID-19 Genomics UK (COG-UK) Consortium	Cordelia Langford; David K. Jackson; Dominic Kwiatkowski; Ewan Harrison; Ian Johnston; John Sillitoe on behalf of the Wellcome Sanger Institute COVID-19 Surveillance Team; John Sillitoe on behalf of the Wellcome Sanger Institute COVID-19 Surveillance Team (http://www.sanger.ac.uk/covid-team); Roberto Amato; Sonia Goncalves; The Lighthouse Lab in Milton Keynes and Alex Alderton
EPI_ISL_598250, EPI_ISL_598251, EPI_ISL_598252, EPI_ISL_598253, EPI_ISL_598254, EPI_ISL_598256, EPI_ISL_598257, EPI_ISL_598258, EPI_ISL_598259, EPI_ISL_598260, EPI_ISL_598261, EPI_ISL_598262, EPI_ISL_598263, EPI_ISL_598264, EPI_ISL_598265, EPI_ISL_598266, EPI_ISL_598267, EPI_ISL_598268, EPI_ISL_598270, EPI_ISL_598271, EPI_ISL_598272, EPI_ISL_598273, EPI_ISL_598274, EPI_ISL_598275, EPI_ISL_598276, EPI_ISL_598277, EPI_ISL_598278, EPI_ISL_598279, EPI_ISL_598280, EPI_ISL_598281, EPI_ISL_598282, EPI_ISL_598283, EPI_ISL_598284, EPI_ISL_598286, EPI_ISL_598287, EPI_ISL_598288, EPI_ISL_598289, EPI_ISL_598290, EPI_ISL_598291, EPI_ISL_598292, EPI_ISL_598293, EPI_ISL_598294, EPI_ISL_598295, EPI_ISL_598296, EPI_ISL_598297, EPI_ISL_598298, EPI_ISL_598299, EPI_ISL_598300, EPI_ISL_598301, EPI_ISL_598302, EPI_ISL_598303, EPI_ISL_598304, EPI_ISL_598306, EPI_ISL_598307, EPI_ISL_598308, EPI_ISL_598309, EPI_ISL_598310, EPI_ISL_598311, EPI_ISL_598313, EPI_ISL_598314, EPI_ISL_598315, EPI_ISL_598316, EPI_ISL_598317, EPI_ISL_598318, EPI_ISL_598319, EPI_ISL_598320, EPI_ISL_598321, EPI_ISL_598322, EPI_ISL_598323, EPI_ISL_598324, EPI_ISL_598325, EPI_ISL_598326, EPI_ISL_598327, EPI_ISL_598328, EPI_ISL_598329, EPI_ISL_598332, EPI_ISL_598333, EPI_ISL_598334, EPI_ISL_598335, EPI_ISL_598336, EPI_ISL_598337, EPI_ISL_598338, EPI_ISL_598339, EPI_ISL_598340, EPI_ISL_598341, EPI_ISL_598342, EPI_ISL_598343, EPI_ISL_598344, EPI_ISL_598345, EPI_ISL_598346, EPI_ISL_598347, EPI_ISL_598348, EPI_ISL_598349, EPI_ISL_598350, EPI_ISL_598352, EPI_ISL_598353, EPI_ISL_598354, EPI_ISL_598355, EPI_ISL_598356, EPI_ISL_598357, EPI_ISL_598358, EPI_ISL_598359, EPI_ISL_598360, EPI_ISL_598362, EPI_ISL_598363, EPI_ISL_598365, EPI_ISL_598366, EPI_ISL_598367, EPI_ISL_598369, EPI_ISL_598370, EPI_ISL_598371, EPI_ISL_598372, EPI_ISL_598373, EPI_ISL_598374, EPI_ISL_598375, EPI_ISL_598376, EPI_ISL_598377, EPI_ISL_598378, EPI_ISL_598379, EPI_ISL_598380, EPI_ISL_598381, EPI_ISL_598382, EPI_ISL_598383, EPI_ISL_598385, EPI_ISL_598386, EPI_ISL_598387, EPI_ISL_598389, EPI_ISL_598390, EPI_ISL_598391, EPI_ISL_598392, EPI_ISL_598393, EPI_ISL_598394, EPI_ISL_598395, EPI_ISL_598396, EPI_ISL_598397, EPI_ISL_598398, EPI_ISL_598399, EPI_ISL_598400, EPI_ISL_598401, EPI_ISL_598402, EPI_ISL_598403, EPI_ISL_598404, EPI_ISL_598405, EPI_ISL_598406, EPI_ISL_598407, EPI_ISL_598409, EPI_ISL_598410, EPI_ISL_598411, EPI_ISL_598412, EPI_ISL_598413, EPI_ISL_598414, EPI_ISL_598415, EPI_ISL_598416, EPI_ISL_598417, EPI_ISL_598418, EPI_ISL_598419, EPI_ISL_598420, EPI_ISL_598421, EPI_ISL_598422, EPI_ISL_598423, EPI_ISL_598424, EPI_ISL_598425, EPI_ISL_598426, EPI_ISL_598427, EPI_ISL_598428, EPI_ISL_598429, EPI_ISL_598430, EPI_ISL_598431, EPI_ISL_598432, EPI_ISL_598434, EPI_ISL_598435, EPI_ISL_598437, EPI_ISL_598438, EPI_ISL_598439, EPI_ISL_598440, EPI_ISL_598441, EPI_ISL_598442, EPI_ISL_598443, EPI_ISL_598444, EPI_ISL_598445, EPI_ISL_598446, EPI_ISL_598447, EPI_ISL_598448, EPI_ISL_598449, EPI_ISL_598450, EPI_ISL_598451, EPI_ISL_598452, EPI_ISL_598453, EPI_ISL_598454, EPI_ISL_598455, EPI_ISL_598456, EPI_ISL_598457, EPI_ISL_598458, EPI_ISL_598459, EPI_ISL_598460, EPI_ISL_598461, EPI_ISL_598462, EPI_ISL_598463, EPI_ISL_598464, EPI_ISL_598465, EPI_ISL_598466, EPI_ISL_598467, EPI_ISL_598468, EPI_ISL_598469, EPI_ISL_598470, EPI_ISL_598471, EPI_ISL_598472, EPI_ISL_598473, EPI_ISL_598474, EPI_ISL_598475, EPI_ISL_598476, EPI_ISL_598477, EPI_ISL_598478, EPI_ISL_598479, EPI_ISL_598480, EPI_ISL_598481, EPI_ISL_598482, EPI_ISL_598483, EPI_ISL_598484, EPI_ISL_598485, EPI_ISL_598486, EPI_ISL_598487, EPI_ISL_598488, EPI_ISL_598489, EPI_ISL_598490, EPI_ISL_598491, EPI_ISL_598492, EPI_ISL_598493, EPI_ISL_598494, EPI_ISL_598495, EPI_ISL_598496, EPI_ISL_598497, EPI_ISL_598498, EPI_ISL_598499, EPI_ISL_598500, EPI_ISL_598501, EPI_ISL_598502, EPI_ISL_598503, EPI_ISL_598505, EPI_ISL_598506, EPI_ISL_598507, EPI_ISL_598508, EPI_ISL_598509, EPI_ISL_598510, EPI_ISL_598511, EPI_ISL_598512, EPI_ISL_598513, EPI_ISL_598515, EPI_ISL_598516, EPI_ISL_598517, EPI_ISL_598518, EPI_ISL_598519, EPI_ISL_598520, EPI_ISL_598521, EPI_ISL_598523, EPI_ISL_598524, EPI_ISL_598525, EPI_ISL_598526, EPI_ISL_598527, EPI_ISL_598528, EPI_ISL_598529, EPI_ISL_598530, EPI_ISL_598531, EPI_ISL_598532, EPI_ISL_598533, EPI_ISL_598534, EPI_ISL_598536, EPI_ISL_598537, EPI_ISL_598538, EPI_ISL_598539, EPI_ISL_598540, EPI_ISL_598541, EPI_ISL_598542, EPI_ISL_598543, EPI_ISL_598544, EPI_ISL_598546, EPI_ISL_598547, EPI_ISL_598548, EPI_ISL_598549, EPI_ISL_598550, EPI_ISL_598551, EPI_ISL_598552, EPI_ISL_598553, EPI_ISL_598554, EPI_ISL_598555, EPI_ISL_598556, EPI_ISL_598557, EPI_ISL_598558, EPI_ISL_598559, EPI_ISL_598560, EPI_ISL_598562, EPI_ISL_598563, EPI_ISL_598565, EPI_ISL_598566, EPI_ISL_598567, EPI_ISL_598569, EPI_ISL_598570, EPI_ISL_598571, EPI_ISL_598573, EPI_ISL_598575, EPI_ISL_598576, EPI_ISL_598577, EPI_ISL_598578, EPI_ISL_598579, EPI_ISL_598580, EPI_ISL_598581, EPI_ISL_598582, EPI_ISL_598583, EPI_ISL_598584, EPI_ISL_598585, EPI_ISL_598586, EPI_ISL_598587, EPI_ISL_598588, EPI_ISL_598589, EPI_ISL_598590, EPI_ISL_598591, EPI_ISL_598592, EPI_ISL_598593, EPI_ISL_598594, EPI_ISL_598595, EPI_ISL_598596, EPI_ISL_598597, EPI_ISL_598598, EPI_ISL_598599, EPI_ISL_598600, EPI_ISL_598601, EPI_ISL_598602, EPI_ISL_598604, EPI_ISL_598605, EPI_ISL_598607, EPI_ISL_598608, EPI_ISL_598609, EPI_ISL_598610, EPI_ISL_598611, EPI_ISL_598612, EPI_ISL_598613, EPI_ISL_598614, EPI_ISL_598615, EPI_ISL_598616, EPI_ISL_598617, EPI_ISL_598618, EPI_ISL_598619, EPI_ISL_598620, EPI_ISL_598621, EPI_ISL_598622, EPI_ISL_598623, EPI_ISL_598624, EPI_ISL_598625, EPI_ISL_598626, EPI_ISL_598627, EPI_ISL_598628, EPI_ISL_598629, EPI_ISL_598630, EPI_ISL_598631, EPI_ISL_598632, EPI_ISL_598633, EPI_ISL_598634, EPI_ISL_598636, EPI_ISL_598637, EPI_ISL_598639, EPI_ISL_598640, EPI_ISL_598641, EPI_ISL_598642, EPI_ISL_598643, EPI_ISL_598644, EPI_ISL_598645, EPI_ISL_598646, EPI_ISL_598647, EPI_ISL_598648, EPI_ISL_598649, EPI_ISL_598650, EPI_ISL_598651, EPI_ISL_598652, EPI_ISL_598653, EPI_ISL_598654, EPI_ISL_598655, EPI_ISL_598656, EPI_ISL_598657, EPI_ISL_598658, EPI_ISL_598659, EPI_ISL_598660, EPI_ISL_598661, EPI_ISL_598662, EPI_ISL_598663, EPI_ISL_598664, EPI_ISL_598665, EPI_ISL_598666, EPI_ISL_598668, EPI_ISL_598669, EPI_ISL_598670, EPI_ISL_598671, EPI_ISL_598672, EPI_ISL_598673, EPI_ISL_598674, EPI_ISL_598675, EPI_ISL_598676, EPI_ISL_598677, EPI_ISL_598678, EPI_ISL_598679, EPI_ISL_598680, EPI_ISL_598681, EPI_ISL_598682, EPI_ISL_598683, EPI_ISL_598684, EPI_ISL_598685, EPI_ISL_598686, EPI_ISL_598687, EPI_ISL_598688, EPI_ISL_598689, EPI_ISL_598690, EPI_ISL_598691, EPI_ISL_598692, EPI_ISL_598693, EPI_ISL_598694, EPI_ISL_598695, EPI_ISL_598696, EPI_ISL_598697, EPI_ISL_598698, EPI_ISL_598699, EPI_ISL_598700, EPI_ISL_598701, EPI_ISL_598702, EPI_ISL_598704, EPI_ISL_598705, EPI_ISL_598706, EPI_ISL_598707, EPI_ISL_598708, EPI_ISL_598709, EPI_ISL_598710, EPI_ISL_598711, EPI_ISL_598712, EPI_ISL_598714, EPI_ISL_598715, EPI_ISL_598716, EPI_ISL_598717, EPI_ISL_598718, EPI_ISL_598719, EPI_ISL_598720, EPI_ISL_598721, EPI_ISL_598722, EPI_ISL_598723, EPI_ISL_598724, EPI_ISL_598725, EPI_ISL_598726, EPI_ISL_598727, EPI_ISL_598728, EPI_ISL_598729, EPI_ISL_598730, EPI_ISL_598731, EPI_ISL_598732, EPI_ISL_598733, EPI_ISL_598734, EPI_ISL_598735, EPI_ISL_598736, EPI_ISL_598737, EPI_ISL_598738, EPI_ISL_598739, EPI_ISL_598740, EPI_ISL_598741, EPI_ISL_598742, EPI_ISL_598743, EPI_ISL_598744, EPI_ISL_598745, EPI_ISL_598747, EPI_ISL_598748, EPI_ISL_598749, EPI_ISL_598750, EPI_ISL_598752, EPI_ISL_598753, EPI_ISL_598754, EPI_ISL_598755, EPI_ISL_598756, EPI_ISL_598757, EPI_ISL_598758, EPI_ISL_598760, EPI_ISL_598761, EPI_ISL_598763, EPI_ISL_598764, EPI_ISL_598767, EPI_ISL_598768, EPI_ISL_598769, EPI_ISL_598770, EPI_ISL_598771, EPI_ISL_598772, EPI_ISL_598773, EPI_ISL_598774, EPI_ISL_598775, EPI_ISL_598776, EPI_ISL_598777, EPI_ISL_598778, EPI_ISL_598779, EPI_ISL_598780, EPI_ISL_598781, EPI_ISL_598782, EPI_ISL_598783, EPI_ISL_598784, EPI_ISL_598785, EPI_ISL_598786, EPI_ISL_598787, EPI_ISL_598788, EPI_ISL_598789, EPI_ISL_598790, EPI_ISL_598791, EPI_ISL_598792, EPI_ISL_598793, EPI_ISL_598794, EPI_ISL_598795, EPI_ISL_598796, EPI_ISL_598797, EPI_ISL_598798, EPI_ISL_598799, EPI_ISL_598800, EPI_ISL_598801, EPI_ISL_598802, EPI_ISL_598803, EPI_ISL_598805, EPI_ISL_598806, EPI_ISL_598807, EPI_ISL_598808, EPI_ISL_598809, EPI_ISL_598810, EPI_ISL_598811, EPI_ISL_598812, EPI_ISL_598813, EPI_ISL_598814, EPI_ISL_598815, EPI_ISL_598816, EPI_ISL_598818, EPI_ISL_598819, EPI_ISL_598820, EPI_ISL_598822, EPI_ISL_609219, EPI_ISL_609220, EPI_ISL_609221, EPI_ISL_609222, EPI_ISL_609223, EPI_ISL_609224, EPI_ISL_609225, EPI_ISL_609226, EPI_ISL_609227, EPI_ISL_609228, EPI_ISL_609229, EPI_ISL_609231, EPI_ISL_609232, EPI_ISL_609233, EPI_ISL_609234, EPI_ISL_609235, EPI_ISL_609236, EPI_ISL_609237, EPI_ISL_609238, EPI_ISL_609239, EPI_ISL_609240, EPI_ISL_609241, EPI_ISL_609242, EPI_ISL_609243, EPI_ISL_609244, EPI_ISL_609245, EPI_ISL_609247, EPI_ISL_609248, EPI_ISL_609249, EPI_ISL_609250, EPI_ISL_609251, EPI_ISL_609252, EPI_ISL_609253, EPI_ISL_609254, EPI_ISL_609255, EPI_ISL_609256, EPI_ISL_609257, EPI_ISL_609258, EPI_ISL_609259, EPI_ISL_609260, EPI_ISL_609261, EPI_ISL_609262, EPI_ISL_609263, EPI_ISL_609264, EPI_ISL_609265, EPI_ISL_609266, EPI_ISL_609268, EPI_ISL_609269, EPI_ISL_609270, EPI_ISL_609271, EPI_ISL_609272, EPI_ISL_609273, EPI_ISL_609274, EPI_ISL_609275, EPI_ISL_609276, EPI_ISL_609277, EPI_ISL_609278, EPI_ISL_609279, EPI_ISL_609280, EPI_ISL_609281, EPI_ISL_609282, EPI_ISL_609283, EPI_ISL_609284, EPI_ISL_609285, EPI_ISL_609287, EPI_ISL_609288, EPI_ISL_609290, EPI_ISL_609291, EPI_ISL_609292, EPI_ISL_609293, EPI_ISL_609294, EPI_ISL_609295, EPI_ISL_609296, EPI_ISL_609297, EPI_ISL_609298, EPI_ISL_609299, EPI_ISL_609300, EPI_ISL_609301, EPI_ISL_609303, EPI_ISL_609304, EPI_ISL_609305, EPI_ISL_609306, EPI_ISL_609307, EPI_ISL_629591, EPI_ISL_630322, EPI_ISL_634418			
see above	Lighthouse Lab in Milton Keynes	Wellcome Sanger Institute for the COVID-19 Genomics UK (COG-UK) consortium	Cordelia Langford; David K. Jackson; Dominic Kwiatkowski; Ewan Harrison; Ian Johnston; John Sillitoe on behalf of the Wellcome Sanger Institute COVID-19 Surveillance Team; John Sillitoe on behalf of the Wellcome Sanger Institute COVID-19 Surveillance Team (http://www.sanger.ac.uk/covid-team); Roberto Amato; Sonia Goncalves; The Lighthouse Lab in Milton Keynes and Alex Alderton
EPI_ISL_595149	Quadram Institute Bioscience	COVID-19 Genomics UK (COG-UK) Consortium	Alexander J Trotter; Alison E. Mather; Alp Aydin; Ana P. Tedim; Anastasia Kolyva; Andrew Bell; Andrew J. Page; Claire Stuart; Dave J. Baker; Gemma L. Kay; John Wain; Justin O'Grady; Leonardo de Oliveira Martins; Lizzie Meadows; Maria Diaz; Mark Webber; Muhammed Yasir; Nabil-Fareed Alikhan; Ngozi Elumogo; Nicholas M. Thomson; Rachael Stanley; Rachel Gilroy; Reenesh Prakash; Samir Dervisevic; Samuel Bloomfield; Steven Rudder; Thanh Le-Viet
EPI_ISL_613205, EPI_ISL_613215, EPI_ISL_613274, EPI_ISL_626788, EPI_ISL_639183, EPI_ISL_639323	Wales Specialist Virology Centre Sequencing lab: Pathogen Genomics Unit	COVID-19 Genomics UK (COG-UK) Consortium	Alec Birchley; Alexander Adams; Amy Gaskin; Angela Marchbank; Bree Gatica-Wilcox; Catherine Moore; Jason Coombes; Joanne Watkins; Joel Southgate; Johnathan Evans; Laura Gifford; Lauren Gilbert; Lee Graham; Malorie Perry; Matthew Bull; Nicole Pacchiarini; Sally Corden; Sara Kumziene-Summerhayes; Sara Rey; Sarah Taylor; Simon Cottrell; Sophie Jones; Tom Connor
