# Supplementary material for: Intrahost SARS-CoV-2 k-mer Identification Method (iSKIM) for Rapid Detection of Mutations of Concern Reveals Emergence of Global Mutation Patterns
Source: Viruses. 2022 Sep 27;14(10):2128. doi: 10.3390/v14102128 (PMC9609618; doi:10.3390/v14102128)
Supplement: Supplementary file 1 [file viruses-14-02128-s001.zip › Supplementary_Files/TableS5.pdf]

We gratefully acknowledge the following Authors from the Originating laboratories responsible for obtaining the specimens, as well as the Submitting laboratories where the genome data were generated and shared via GISAID, on which this research is based.

All Submitters of data may be contacted directly via www.gisaid.org

Authors are sorted alphabetically.

Acknowledgement EPI_SET Identifier: EPI_SET_20220218oz

Accession ID	Originating Laboratory	Submitting Laboratory	Authors
EPI_ISL_567538, EPI_ISL_567580, EPI_ISL_567699, EPI_ISL_567829	Lighthouse Lab in Alderley Park	Wellcome Sanger Institute for the COVID-19 Genomics UK (COG-UK) consortium	Cordelia Langford; David K. Jackson; Dominic Kwiatkowski; Ewan Harrison; Ian Johnston; Jacquelyn Wynn; John Sillitoe on behalf of the Wellcome Sanger Institute COVID-19 Surveillance Team; Mairead Hyland; Roberto Amato; Sonia Goncalves; The Lighthouse Lab in Alderley Park and Alex Alderton
EPI_ISL_566610	Lighthouse Lab in Cambridge	Wellcome Sanger Institute for the COVID-19 Genomics UK (COG-UK) consortium	Cordelia Langford; David K. Jackson; Dominic Kwiatkowski; Ewan Harrison; Ian Johnston; John Sillitoe on behalf of the Wellcome Sanger Institute COVID-19 Surveillance Team; Rob Howes; Roberto Amato; Sonia Goncalves; The Lighthouse Lab in Cambridge and Alex Alderton
EPI_ISL_602090	Lighthouse Lab in Glasgow	Wellcome Sanger Institute for the COVID-19 Genomics UK (COG-UK) Consortium	Anna Dominiczak and Alex Alderton; Carol Clugston; Cordelia Langford; David Gray; David K. Jackson; Dominic Kwiatkowski; Ewan Harrison; Harper VanSteenhouse; Ian Johnston; John Sillitoe on behalf of the Wellcome Sanger Institute COVID-19 Surveillance Team; Roberto Amato; Sonia Goncalves; Yumi Kasai
EPI_ISL_549554, EPI_ISL_549594, EPI_ISL_549801, EPI_ISL_567311, EPI_ISL_567457, EPI_ISL_568244, EPI_ISL_568250, EPI_ISL_568273, EPI_ISL_568278, EPI_ISL_580886, EPI_ISL_580947, EPI_ISL_580977, EPI_ISL_581080, EPI_ISL_581194, EPI_ISL_588234, EPI_ISL_588727, EPI_ISL_588741, EPI_ISL_588748, EPI_ISL_588761, EPI_ISL_588805, EPI_ISL_588971, EPI_ISL_590177, EPI_ISL_590315, EPI_ISL_590670, EPI_ISL_599489, EPI_ISL_599504, EPI_ISL_599511, EPI_ISL_599561, EPI_ISL_599575, EPI_ISL_599633, EPI_ISL_599653, EPI_ISL_599686, EPI_ISL_599714, EPI_ISL_599747, EPI_ISL_600575, EPI_ISL_600587, EPI_ISL_600855, EPI_ISL_600872, EPI_ISL_601104, EPI_ISL_601108, EPI_ISL_601127, EPI_ISL_601168, EPI_ISL_601175, EPI_ISL_601206, EPI_ISL_601208, EPI_ISL_601334, EPI_ISL_601357			
see above	Lighthouse Lab in Glasgow	Wellcome Sanger Institute for the COVID-19 Genomics UK (COG-UK) consortium	Anna Dominiczak and Alex Alderton; Carol Clugston; Cordelia Langford; David Gray; David K. Jackson; Dominic Kwiatkowski; Ewan Harrison; Harper VanSteenhouse; Ian Johnston; John Sillitoe on behalf of the Wellcome Sanger Institute COVID-19 Surveillance Team; John Sillitoe on behalf of the Wellcome Sanger Institute COVID-19 Surveillance Team (http://www.sanger.ac.uk/covid-team); Roberto Amato; Sonia Goncalves; Yumi Kasai
EPI_ISL_549988, EPI_ISL_550150, EPI_ISL_566436, EPI_ISL_566447, EPI_ISL_566452, EPI_ISL_566471, EPI_ISL_566484, EPI_ISL_566506, EPI_ISL_566570, EPI_ISL_566728, EPI_ISL_580915, EPI_ISL_581342, EPI_ISL_599928, EPI_ISL_599964, EPI_ISL_600016			
see above	Lighthouse Lab in Milton Keynes	Wellcome Sanger Institute for the COVID-19 Genomics UK (COG-UK) consortium	Cordelia Langford; David K. Jackson; Dominic Kwiatkowski; Ewan Harrison; Ian Johnston; John Sillitoe on behalf of the Wellcome Sanger Institute COVID-19 Surveillance Team; John Sillitoe on behalf of the Wellcome Sanger Institute COVID-19 Surveillance Team (http://www.sanger.ac.uk/covid-team); Roberto Amato; Sonia Goncalves; The Lighthouse Lab in Milton Keynes and Alex Alderton
