# Supplementary material for: Intrahost SARS-CoV-2 k-mer Identification Method (iSKIM) for Rapid Detection of Mutations of Concern Reveals Emergence of Global Mutation Patterns
Source: Viruses. 2022 Sep 27;14(10):2128. doi: 10.3390/v14102128 (PMC9609618; doi:10.3390/v14102128)
Supplement: Supplementary file 1 [file viruses-14-02128-s001.zip › Supplementary_Files/TableS6.pdf]

SUPPLEMENTAL TABLE

Data Availability

GISAID Identifier:	EPI_SET_20220603uy
DOI:	https://doi.org/10.55876/gis8.220603uy

All genome sequences and associated metadata in this dataset are published in GISAID's EpiCoV database. To view the contributors of each individual sequence with details such as accession number, Virus name, Collection date, Originating Lab and Submitting Lab and the list of Authors, visit https://doi.org/10.55876/gis8.220603uy

Data Snapshot

- EPI_SET_20220603uy is composed of 3,242 individual genome sequences.
- The collection dates range from 2019-12-26 to 2022-01-07;
- Data were collected in 192 countries and territories;
- All sequences in this dataset are compared relative to hCoV-19/Wuhan/WIV04/2019 (WIV04), the official reference sequence employed by GISAID (EPI_ISL_402124). Learn more at https://gisaid.org/WIV04.
