# Supplementary figures and images for: Intrahost SARS-CoV-2 k-mer Identification Method (iSKIM) for Rapid Detection of Mutations of Concern Reveals Emergence of Global Mutation Patterns
Source: Viruses. 2022 Sep 27;14(10):2128. doi: 10.3390/v14102128 (PMC9609618; doi:10.3390/v14102128)

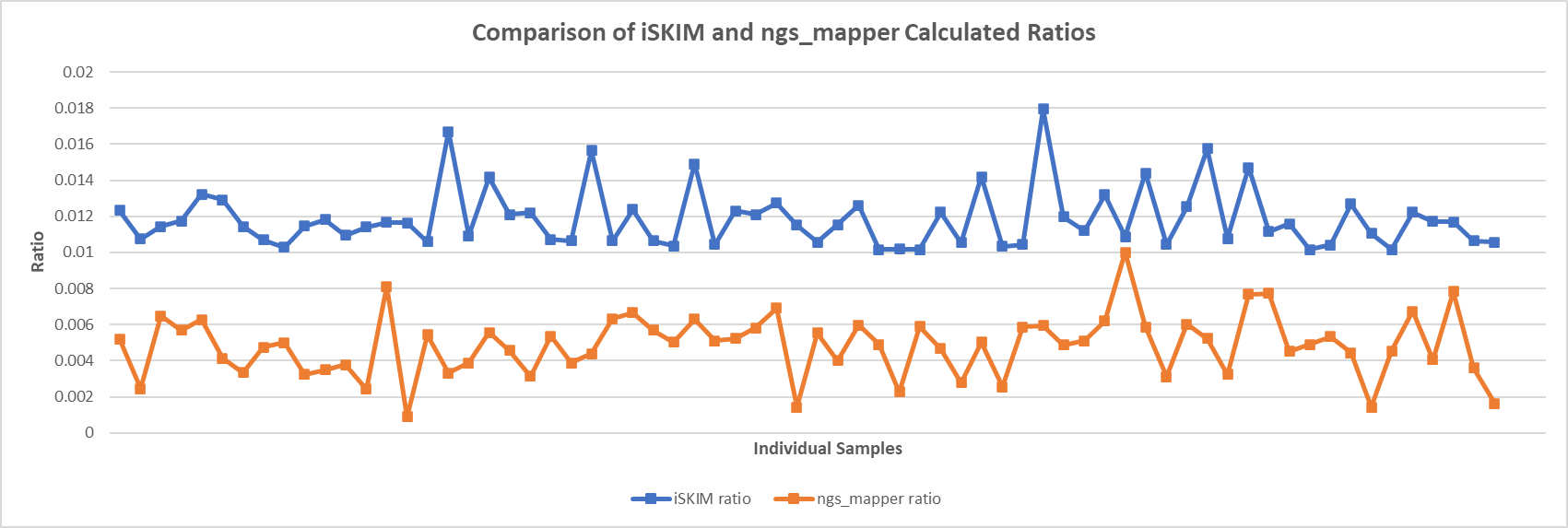

Supplement: Supplementary file 1 [file viruses-14-02128-s001.zip › Supplementary_Files/Figure_S2.png]

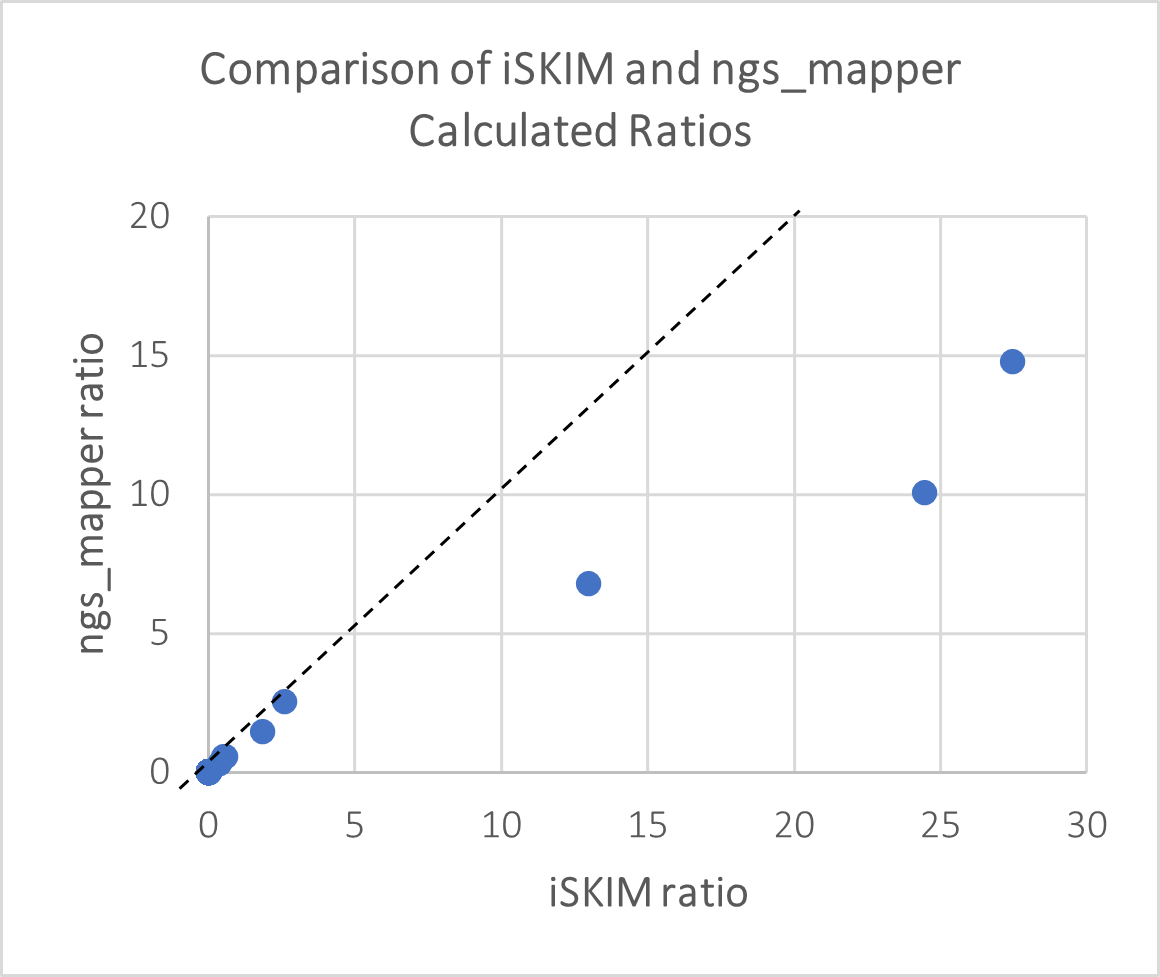

Supplement: Supplementary file 1 [file viruses-14-02128-s001.zip › Supplementary_Files/Figure_S3.png]

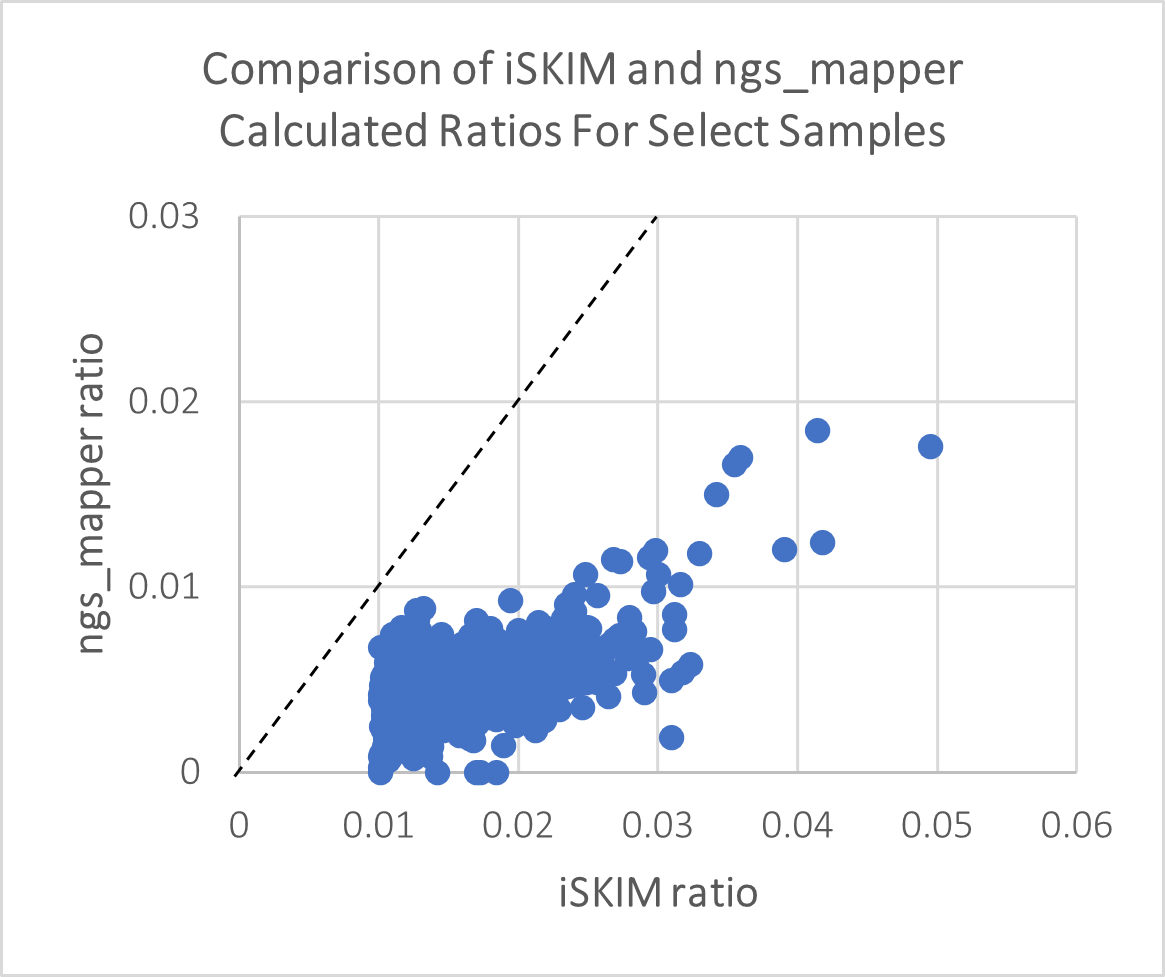

Supplement: Supplementary file 1 [file viruses-14-02128-s001.zip › Supplementary_Files/Figure_S4.png]

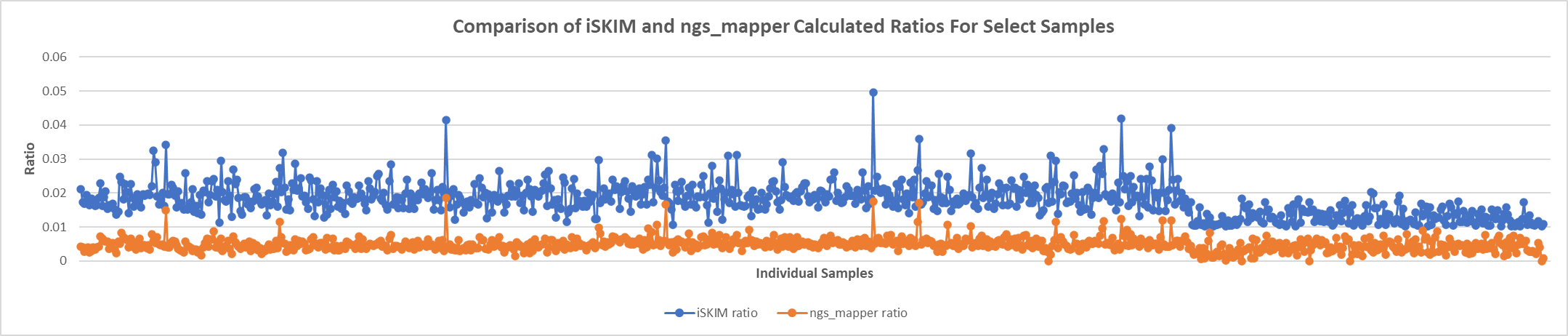

Supplement: Supplementary file 1 [file viruses-14-02128-s001.zip › Supplementary_Files/Figure_S5.png]

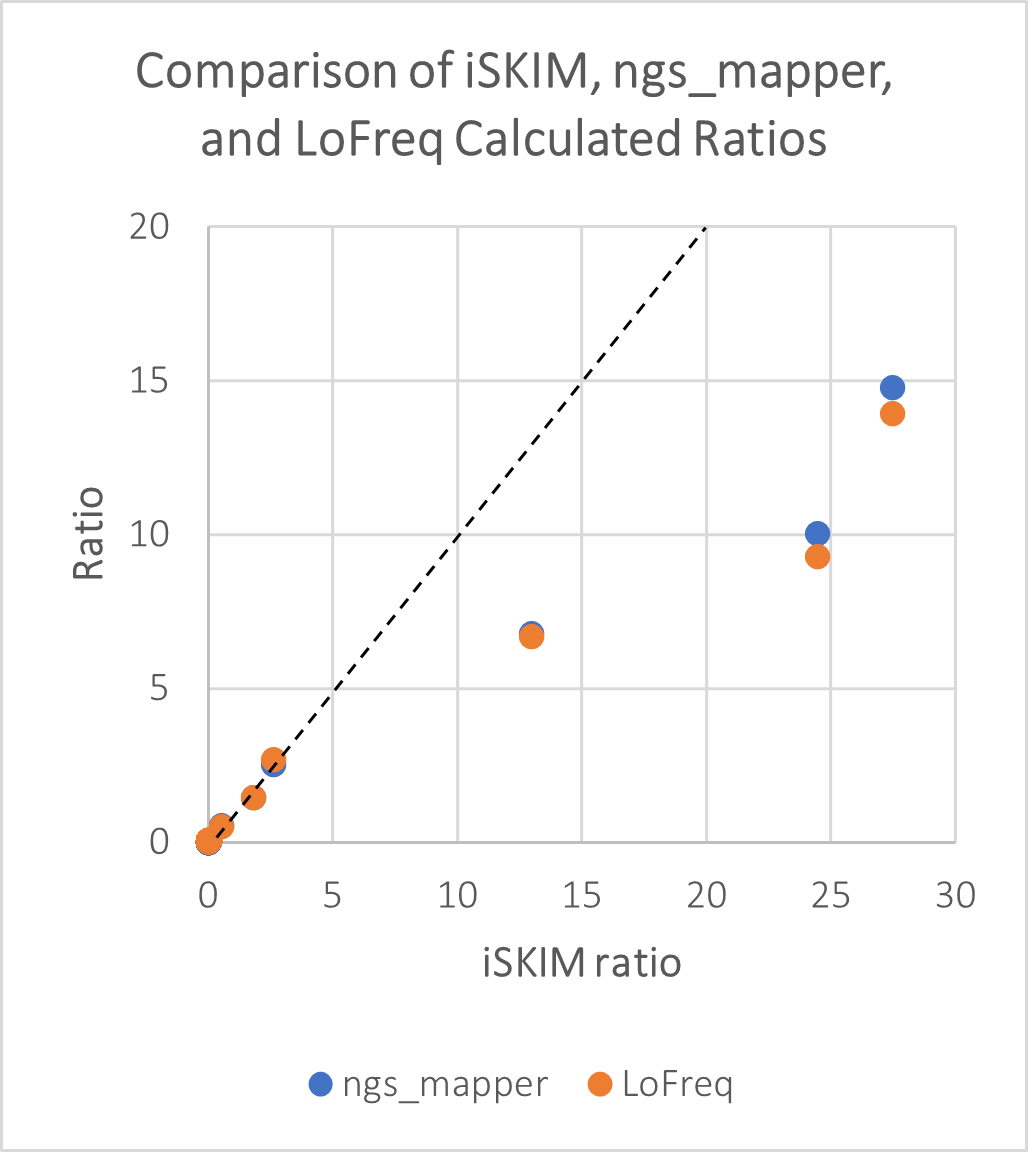

Supplement: Supplementary file 1 [file viruses-14-02128-s001.zip › Supplementary_Files/Figure_S6.png]

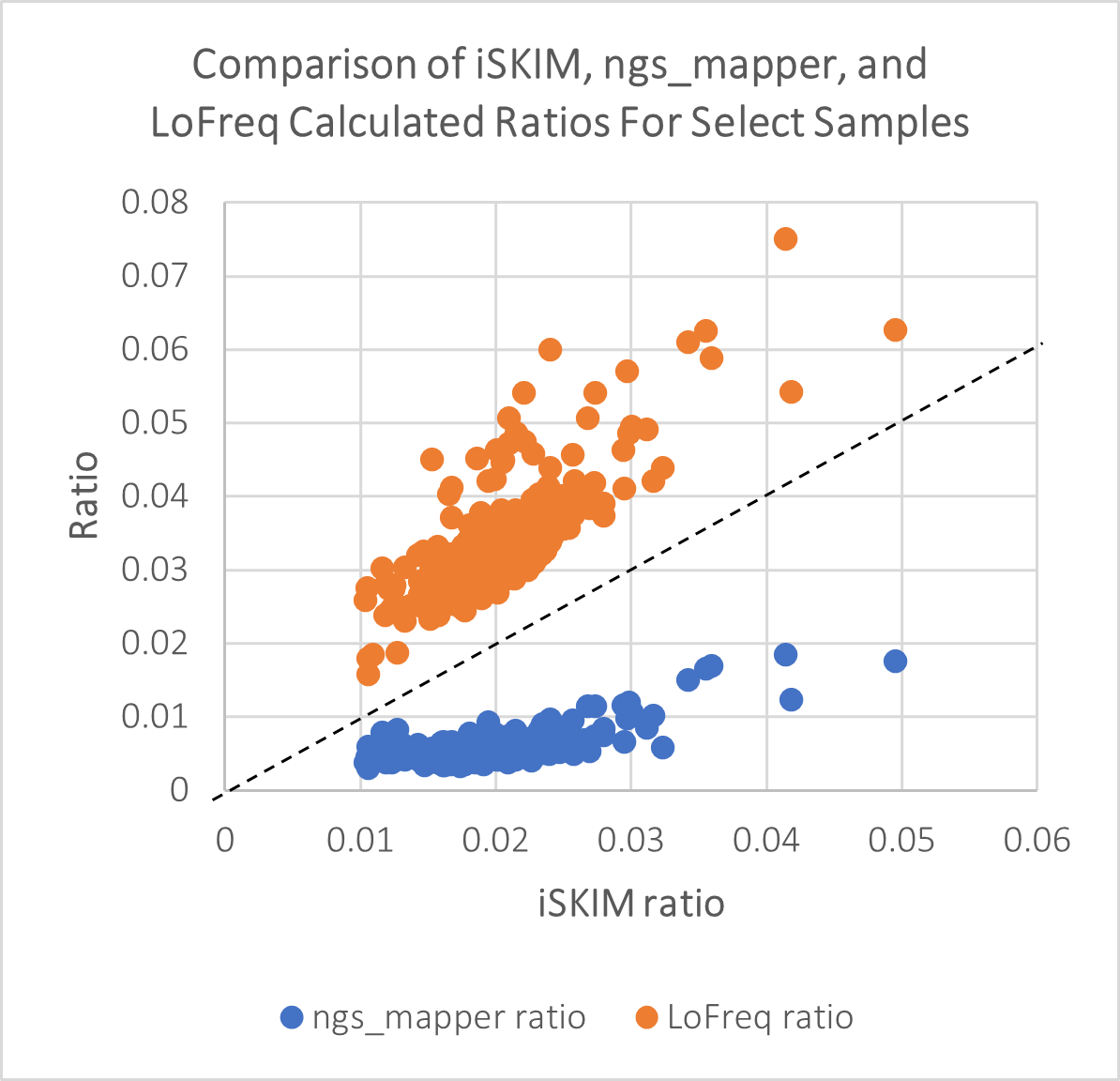

Supplement: Supplementary file 1 [file viruses-14-02128-s001.zip › Supplementary_Files/FIgure_S7.png]

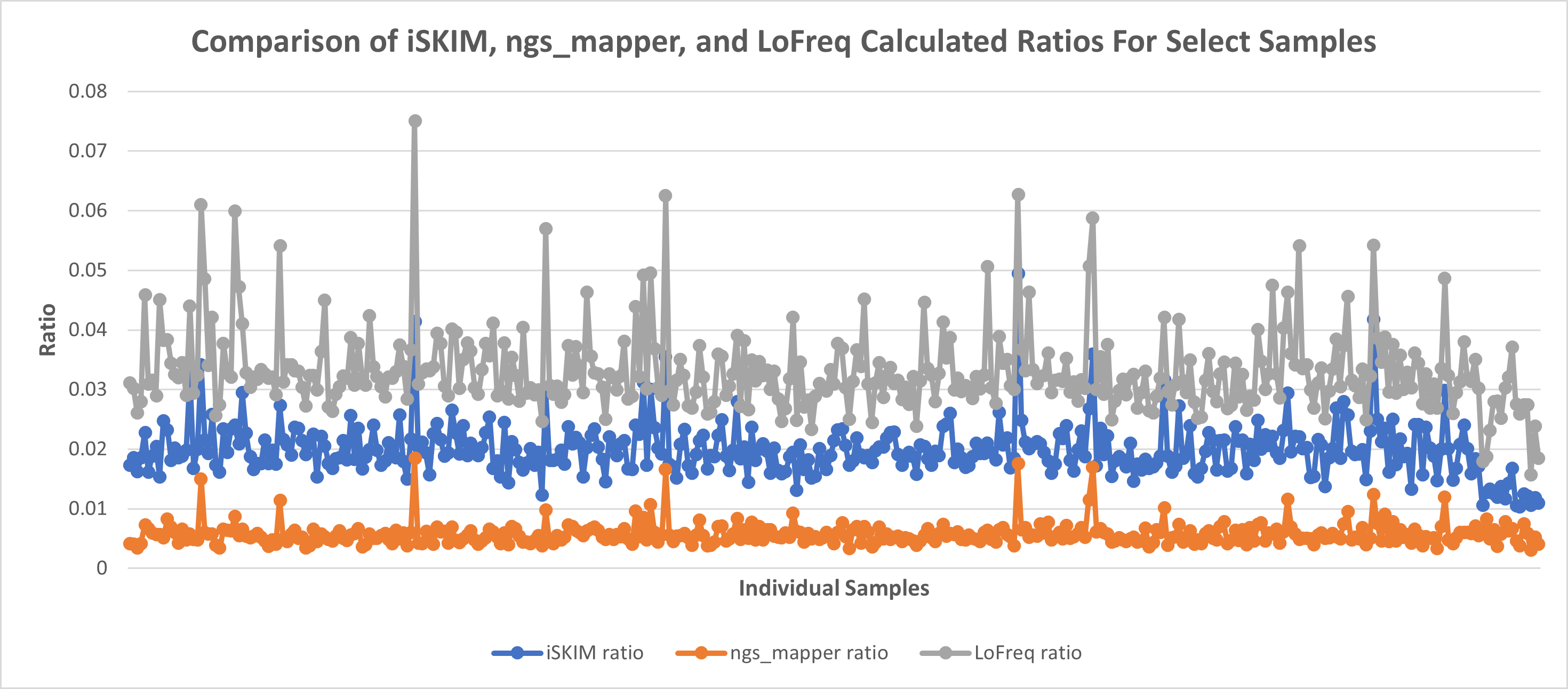

Supplement: Supplementary file 1 [file viruses-14-02128-s001.zip › Supplementary_Files/Figure_S8.png]
